# Anaerobic Digested Wastewater CO_2_ Sequestration Using a Biophotocatalytic System with a Magnetized Photocatalyst (Fe-TiO_2_)

**DOI:** 10.3390/molecules27165213

**Published:** 2022-08-16

**Authors:** Emmanuel Kweinor Tetteh, Gloria Amo-Duodu, Sudesh Rathilal

**Affiliations:** Green Engineering Research Group, Faculty of Engineering and the Built Environment, Department of Chemical Engineering, Durban University of Technology, Steve Biko Campus (S4 Level 1) Box 1334, Durban 4000, South Africa

**Keywords:** anaerobic digestion, bioenergy, bio photocatalysis, biogas, magnetic nanomaterials, hydrolysis, wastewater

## Abstract

This study presents a biophotocatalytic system as a sustainable technology for the recovery of clean water and renewable energy from wastewater, thereby providing a unique opportunity to drive industrialization and global sustainable development throughputs. Herein, inhouse magnetized photocatalyst (Fe-TiO_2_) with surface area 62.73 m^2^/g synthesized via co-precipitation, was hypothesized to hasten an up-flow anaerobic sludge blanket (UASB) reactor for the treatment of local South Africa municipality wastewater with the benefit of high-quality biogas production. A lab scale UASB process with a working volume of 5 L coupled with two UV-lights (T8 blacklight-blue tube, 365 nm, 18 W) was operated batchwise under mesophilic conditions for the period of 30 days with a constant organic load charge of 2.76 kg COD/m^3^. d. This biophotocatalytic system performance was investigated and compared with and without the Fe-TiO_2_ charge (2–6 g) with respect to effluent quality, biogas production and CO_2_ methanation. Using chemical oxygen demand (COD) measured as the degree of degradation of the pollutants, the best efficiency of 93% COD removal was achieved by a 4 g Fe-TiO_2_ charge at 14 days and pH of 7.13, as compared to zero charge where only 49.6% degradation was achieved. Under the same charge, cumulative biogas and methane content of 1500 mL/g COD.d and 85% were respectively attained as compared with the control with 400 mL/g COD.d and 65% methane content. Also, the energy produced can be used to offset the energy utilized by the UV-light for the wastewater abatement and other limitations of photocatalysis. The BP system was found to be an eco-friendly and cost-effective technology to be explored in water treatment settings.

## 1. Introduction

In recent years, the industrial revolution has been exploiting resources for manufacturing and consumption with no provisions for waste reuse or economic regeneration [1]. Herein, the world’s linear economy is shown to be at a critical point of fossil fuel depletion with environmental challenges [2,3]. Against this background, it is time to consider waste resource utilization towards a sustainable circular economy [4,5,6]. In essence, biogas production from wastewater could help to address the global economy, as well as ecological and CO_2_ anthropogenic emission concerns [7,8]. As a result, implementing a CO_2_-based circular economy could help alleviate CO_2_ emissions and pave the way for future resource independence [4,6]. Therefore, developing biological CO_2_ sequestration technology with environmental benefits becomes very critical.

Consequentially, industrial wastewater containing refractory chemicals (e.g., synthetic dyes, phenolics, emerging contaminants) has had a detrimental effect on the ecosystem and human health and has hastened the global debate on clean water and sanitation [3,9,10,11]. However, the conventional wastewater technologies (coagulation, chlorination, biodegradation) are not cost-effective nor efficient in removing recalcitrant pollutants from the wastewater [10,12,13,14,15]. This warrants new technological solutions for effective, economical, and environmentally friendly wastewater treatment for reuse and sustainability. Standalone treatments are classically limited; for instance, anaerobic digestion (AD) has been a biological tool for producing biogas from wastewater treatment [4,16,17]. However, due to self-inhibition at high pollutant concentrations, accumulation of intermediate metabolites and slow kinetics, conventional AD is generally ineffective in treating refractory pollutants [16,18,19,20]. In addressing this setback, a combination of biocatalysis with photocatalysis is receiving significant interest due to their single treatment inefficiency or high cost. Rodríguez-Couto [21] reported on biophotodegradation of emerging pollutants from the wastewater as a promising technology. Molla, et al. [22] also reviewed recent trends in nanomaterials and nanocomposites for environmental applications. In addition, engineered magnetized photocatalysts could easily be separated from a solution by an external magnet after the biophotocatalytic activity [23,24,25]. Herein, in a biophotocatalytic sequential system, a number of limitations can still be observed, including high electricity consumption, high building costs, and high operating expenses [21]. The aforementioned drawbacks can be overcome by combining photochemical and biological techniques, resulting in photocatalysis and biodegradation occurring simultaneously [26]. This method will enable the treatment of wastewater containing recalcitrant organic contaminants to have synergy.

Therefore, in this study, an up-flow anaerobic sludge blanket (UASB) reactor was coupled with UV lights as a biophotocatalytic (BP) system was investigated for a local South Africa wastewater treatment. To evaluate its efficacy for rapid degradation of the contaminants, along with biogas production, the application of a previously engineered magnetized photocatalyst (Fe-TiO_2_) was introduced into the BP system. In addition, suitable kinetic models and the price of the energy that the bioenergy produced were assessed.

## 2. Results

**Figure 1:**
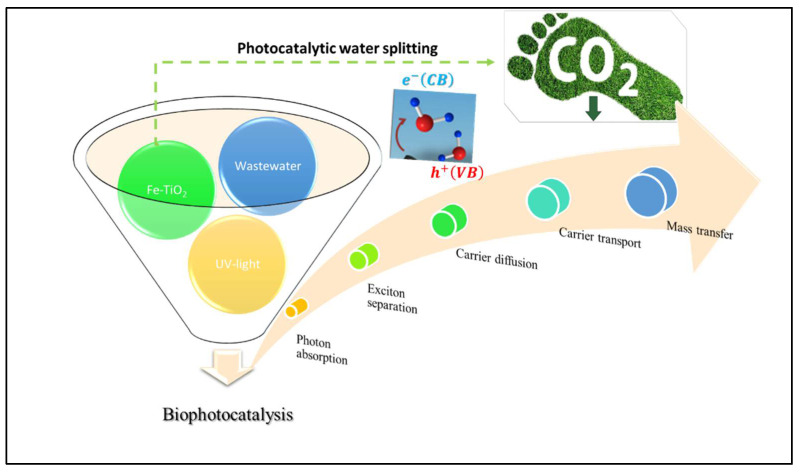
A schematic diagram of biophotocatalysis.

In this study, biocatalysts (microorganisms or enzymes) and a photochemical catalyst (Fe-TiO_2_) were explored to convert wastewater from eco-destructive to bioenergy resource-saving with ecological benefits. Brunauer–Emmett–Teller (BET) Micrometric analyser (TriStar II Plus, Norcross, GA, USA) revealed the inhouse Fe-TiO_2_ had high surface area (62.73 m^2^/g) as compared to the conventional TiO_2_ (25.7 m^2^/g). This transformation and surface area modification were due to the incorporation of the Fe_3_O_4_ (27.6 m^2^/g). The phase identification results by X-ray diffraction (XRD) (Bruker AXS, D8 Advance, Germany), coupled with PANalytical software (Empyrean, PRO MPD, Netherlands), also revealed the crystalline structure of maghemite (γ-Fe_2_O_3_), magnetite (Fe_3_O_4_), and anatase with respective files of JCPDS 00-070-2091, JCPDS00-019-0629 and JCPDS 00-021-0428. The combination of photons (UV-light) and engineered magnetized photocatalyst (Fe-TiO_2_) with the ability to use UV-light energy was introduced into a BP system. This concept, as shown in Figure 1, originates from photosynthesis and the water-molecule splitting technique as an option for renewable-energy production [26]. The Fe-TiO_2_ electrons (h^+^ and e^−^), once illuminated by the UV-light, becomes excited as charged carriers, which are then transported via diffusion mass transfer. In the bio-photo sequence, the biodegradable pollutants were treated biologically, whereas the nonbiodegradable pollutants were photodegraded [21,26]. Also, the kinetic study, as well as the biogas output and composition, were monitored. In addition, to elucidate the business model of the BP system, the power generated to compensate for the UV-light energy was estimated.

### 2.1. Treatability Efficiency of the BP System

**Figure 2:**
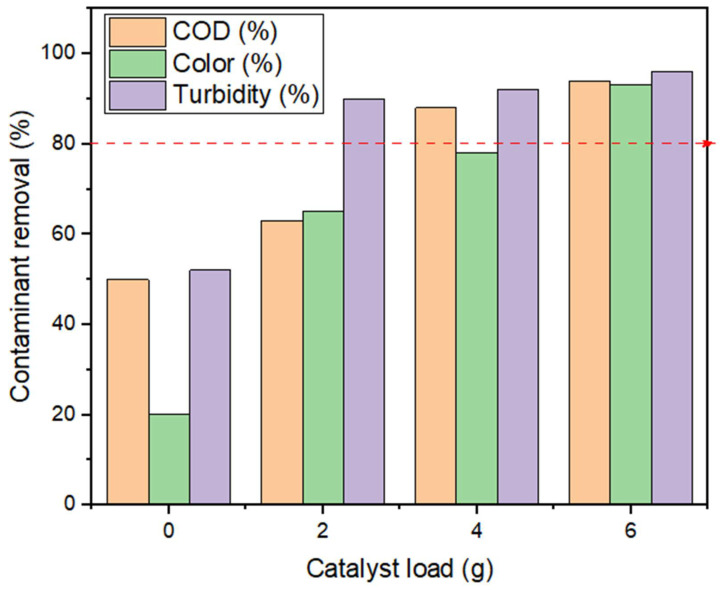
The effect of a weekly catalyst load of Fe-TiO_2_ on contaminant removal by using the biophotocatalytic system.

The wastewater sample was biophotocatalytically treated with the BP system operated at different sludge retention times (SRTs) of 14, 21 and 29 days, representing a weekly dose of 2 g Fe-TiO_2_ for week 2–4. As shown in Figure 2, increasing the catalyst load from 2 g to 6 g Fe-TiO_2_ for the 2–4 weeks increased the contaminant removal in terms of COD, color and turbidity to above 80%. Table 1 presents a summary of the final effluent concentration of other water parameters per the catalyst load charged for a week.

### 2.2. The BP System Biogas Production

**Figure 3:**
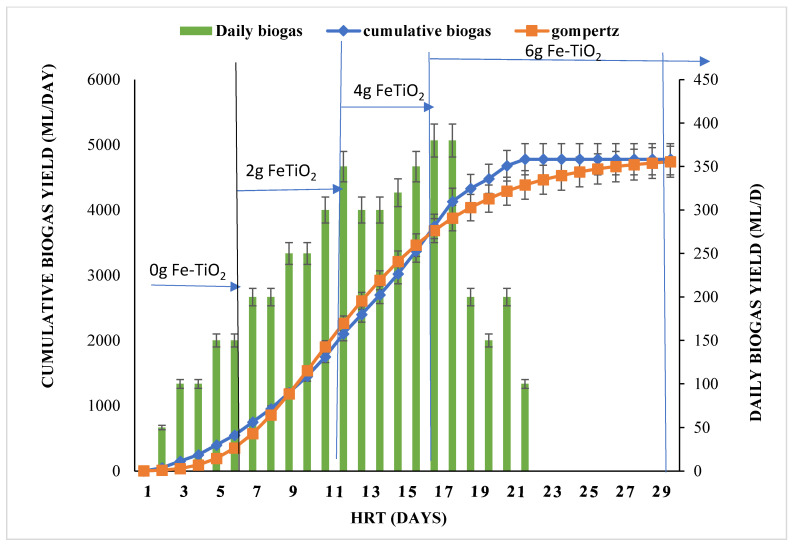
The effect of a weekly catalyst load of Fe-TiO_2_ on biogas production using the biophotocatalytic system for 30 days.

**Figure 4:**
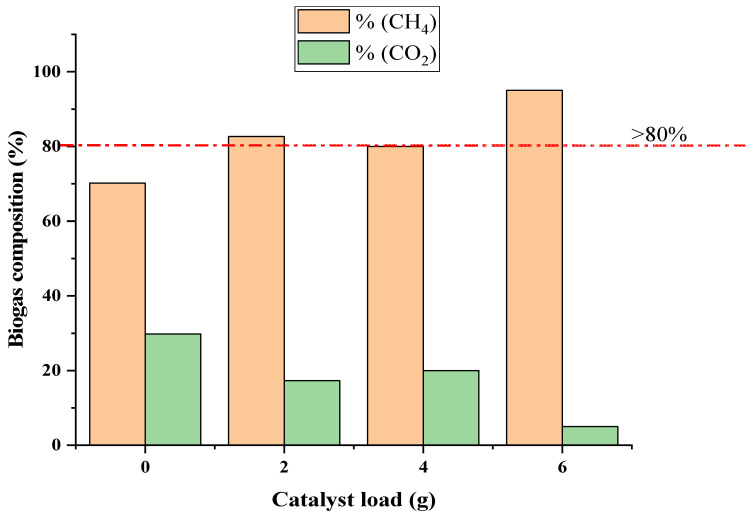
The effect of weekly catalyst load of Fe-TiO_2_ on methane yield using the biophotocatalytic system.

**Figure 5:**
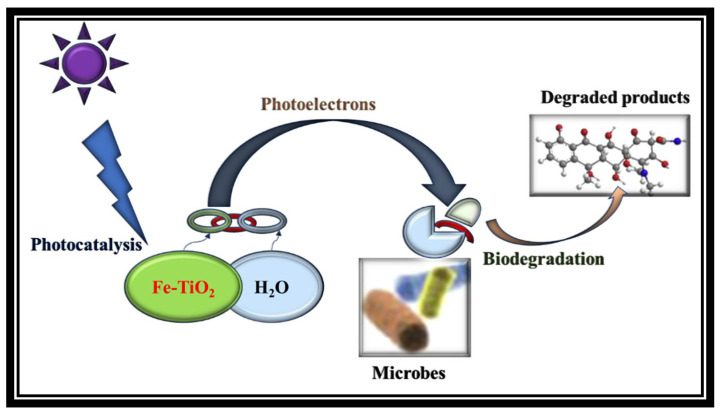
A schematic diagram of the biophotocatalytic system degradation mechanism.

Ideally, AD produces biogas by breaking down high organic molecules of the wastewater, whereas mineralization of the organic molecules via UV photodegradation requires energy. This necessitated the weekly biogas production monitoring and characterization of the BP system. The results obtained are presented in Figure 3, depicting the effect of the catalyst load (Fe-TiO_2_) on biogas production for the 30 days. The cumulative biogas data obtained was fitted on the first order and modified Gompertz kinetic models with information presented in Table 2. The weekly biogas characterized composition with over 80% CH_4_ improvement, as compared to the week with no dosage of the Fe-TiO_2_, is presented in Figure 4. Also, the energy cost from the BP system biogas produced is presented in Table 3. Figure 5 also shows the biophotocatalytic pathway of degrading organic contents of using the Fe-TiO_2_.

## 3. Discussion

### 3.1. Effect of Catalyst Load on Contaminant Reduction

The effect of the catalyst load was conducted ranging from 2 g to 6 g, while keeping other parameters constant with an initial effluent concentration of 2380 mg COD/L of municipality wastewater. Prior to this, a zero charge of the catalyst load was initially conducted as a control to observe the impact of the catalyst load. This was carried out to understand the mechanism of how the catalyst helps with photodegradation of the organic contaminants. As shown in Figure 2, the color removal efficiency was greater than the COD by 5% at a catalyst load of 2 g, implying that there was mineralization of color-causing bonds (chromophores) in the wastewater [27,28]. The best efficiency of COD, color, turbidity and reduction of other organic contaminants (Table 1) was achieved when the reactor had adapted/stabilized in the fourth week (4). Also, the presence of the UV light caused illumination and excitation on the surface of the Fe-TiO_2_, whereby the excited radicals/electrons moved from the catalyst valance band to the conduction band, creating a hole on the surface for easing adsorption of the pollutants [27,29,30]. This elucidates that the Fe-TiO_2_ had potential of electron transfer (Figure 1), whose charge induced separation species (Fe, e^−^, h^+^) by the absorption of the photons. This influenced the degradability of the high organic pollutants (>80% COD removal) at a high catalyst load of 6 g Fe-TiO_2_, as shown in Figure 2.

Furthermore, the Fe-TiO_2_ composite had the tendency to have nutrient rich elements (Fe) with strong photocatalytically induced catalyst (TiO_2_), which influenced the microbial activity (biodegradation). In fact, this mechanism produced active radicals which photocatalytically induced the degradation of the wastewater, while suppressing the oxidation-reduction potential of antibacterial activity [31]. The reaction mechanism, as presented (1–4), shows what the hole and electron species created, reacting with the oxygen or water molecules to produce peroxide or hydroxyl species which in turn act as reactive oxidation reduction agents of the pollutants [31,32].

(1) $$\mathrm{Exposure}\;\mathrm{to}\;\mathrm{UV}-\mathrm{light}\;\left(\mathrm{excitation}\right){\;\mathrm{TiO}}_{2}+\mathrm{hv}\to e^{-}+h^{+}$$

(2) $${\mathrm{Recombination}\;e}^{-}+h^{+}\to \mathrm{hv}$$

(3) $${\mathrm{Superoxide}\;\mathrm{ion}\;\mathrm{radical}\;O}_{2}+{\;e}^{-}\to O_{2}^{*-}$$

(4) $${\mathrm{Hydroxyl}\;\mathrm{radicals}\;\mathrm{OH}}^{-}+h^{+}\to {\mathrm{OH}}^{*}$$

The iron (III) ions of the Fe-TiO_2_ also behave as electrons (5 and 6) and hole traps (7 and 8), which influence the photocatalytic activity via the mass electron transfer.

(5) $${\mathrm{Electron}\;\mathrm{transfer}\;\mathrm{Fe}}^{3+}+{\;e}^{-}\to {\mathrm{Fe}}^{2+}$$

(6) $${\mathrm{Electron}\;\mathrm{transfer}\;\mathrm{Fe}}^{2+}+O_{2}\;\to {\mathrm{Fe}}^{3+}+O_{2}^{*-}$$

(7) $${\mathrm{Hole}\;\mathrm{trap}\;\mathrm{Fe}}^{3+}+h^{+}\to {\mathrm{Fe}}^{4+}$$

(8) $${\mathrm{Hole}\;\mathrm{trap}\;\mathrm{Fe}}^{4+}+{\mathrm{OH}}^{-}\to {\mathrm{Fe}}^{3+}+{\;\mathrm{OH}}^{*}$$

From Table 1, the balanced pH within 7.13 to 7.69, COD (12–115 mg COD/L) and VS/TS ratio < 0.5 proves that there was a degradation of organics. Also, the production of nitrates (TN) and ammonium (NH_4_^+^) during the degradation pathway suggests that there was mineralization of the recalcitrant nitrogen-containing organic compounds, such as TKN, NO_3_-N, and NH_4_^+^ in different proportions [27,30]. Thus, during the reductive pathway, the amount of TN present in the wastewater, at a high oxidation state, produced more unstable intermediates such as TKN and NH_4_^+^ [21]. Likewise, in the lower oxidation state, most of the elemental nitrogen molecules detached resulting in nitrites (NO_3_^−^). Also, the more NH_4_^+^ produced than NO_3_^−^ in this investigation, the more nitrogen atoms elucidated in the wastewater stream had a higher oxidation state and were eliminated via the photocatalysis pathway compared to the biological route [21,27].

### 3.2. Biophotocatalytic CO_2_ Sequestration

As mentioned, anthropogenic CO_2_ emissions are of global concern, whereby wastewater treatment cannot be exempted. Conventionally, the production of biogas from wastewater via AD systems is kinetically slow with the poor quality of both water and biogas warranting a more robust technology. For this purpose, the BP system was employed to investigate the effect of the catalyst load (Fe-TiO_2_) on the CO_2_ sequestration from anaerobically digested biogas for its methane enhancement.

Figure 3 shows the BP system cumulative biogas (5150 mL) obtained for the duration of 30 days with respect to the weekly catalyst load (2–6 g Fe-TiO_2_). The average amount of biogas weekly recorded was 110 mL (zero charge), 270 mL (2 g charge), 285 mL (4 g charge), and 55 mL (6 g charge). It was found that the biogas production increased from the zero-charged catalyst up to the 6 g charged, where there was retardation of the biogas production from the 21st day to the 30th day. This reduction in production might be due to the optimum load of the catalyst (4 g), where the excess catalyst and other intermediate compounds generated inhibited the biogas production. Thus, the microorganism activity was hampered by the high catalyst load from the 21st to the 30th day, which will require a longer period for the microorganisms to adjust and adapt to that condition. Conversely, the highest amount of the catalyst load (6 g) charged increased the methane yield (70% CH_4_) of the zero charge to 95% CH_4_ as depicted in Figure 4.

The modified Gompertz model was selected as best fit (R^2^ = 0.9917), based on the statistical analysis of the cumulative biogas (*p* < 0.05). The weekly addition of the Fe-TiO_2_ at 4 g delivered the highest biogas yields in comparison with others (Figure 3), whereas the addition of Fe-TiO_2_ at 6 g significantly increased the methane yield (Figure 4). This performance could be a result of the presence of Fe and TiO_2_, which served as a direct electron transfer species [33,34], and which facilitated the CO_2_ photoreduction mechanism to generate more methane (>80% CH_4_). Also, the UV-light, together with the catalyst load charged, stimulated the methanogen activity, which resulted in more of the methane being produced. Additionally, the photocatalysis phenomenon generates radical species (OH^−^ and H^+^) which served as a reducing agent [27,33,35], hence converted the CO_2_ to CH_4_.

Generally, degradation of organic content into biogas involves hydrolysis, acidogenesis, acetogenesis and methanogenesis. Notwithstanding, at the hydrolysis and acidogenesis stages, more complex radical ions forming on the surface of the Fe-TiO_2_ prompted the photocatalytic reactions (9–10) by the reactive oxygen species (ROSs) of hole and electron produced (h^+^, e^−^). These radicals then react with dissolved oxygen forming HO_2_* and CO_2_. The reaction then enhances the formation of carbon dioxide and iron (II) oxalate complex (11), which are easy to be decomposed via the methanogenesis phase (methane formation).

(9) $$\mathrm{Fe}-\mathrm{TiO}\overset{\mathrm{hv}}{\to }e^{-}\left(\mathrm{CB}\right)+h^{+}\left(\mathrm{VB}\right)$$

(10) $$h^{+}+{\mathrm{HC}}_{2}O_{4}{}^{-}\to {\mathrm{CO}}_{2}+{\mathrm{COOH}}^{•}$$

(11) $$2{\left[\mathrm{Fe}{\left(C_{2}O_{4}\right)}_{2}\right]}^{3-}\;\overset{\mathrm{hv}}{\to }\;2{\left[\mathrm{Fe}{\left(C_{2}O_{4}\right)}_{2}\right]}^{2-}+C_{2}O_{4}{}^{2-}+2{\mathrm{CO}}_{2}$$

Furthermore, the ROSs further react with dissolved oxygen that provides compounds with an ozonide-like structure, favoring the formation of ring-opening products such as methane [24,27,30]. Methane, which is one of the key components of biogas, involves the conversion of acetate or the decomposition of CO_2_ via hydrogeneration (12–14) by either acetotrophic or hydrogenotrophic microorganisms.

(12) CO2+4H2→CH4+2H2O  ΔG0=−130.7 kJmol

(13) 2CO2+4H2→CH3COOH+2H2O  ΔG0=−104.5 kJmol

(14) CH3COOH→CH4+2H2O  ΔG0=−31 kJmol

Figure 5 shows that there are biochemical pathways for the decomposing CO_2_ in biogas to methane via the biophotodegradation of organic content of wastewater. Herein, the BP system being coupled with UV light ignited the Fe-TiO_2_ (photocatalyst) and indirectly produced the H_2_ source from the hydrolysis of the water molecules splitting [26]. This H_2_ is extremely important to the overall process of biophotocatalytic degradation because it ensures that the biochemical reaction is carried out in a state of equilibrium [27,28]. Through the activity of methanogenic microorganisms, this in situ source of H_2_ in the BP system reacts with CO_2_, which results in the production of CH_4_. The hydrogenotrophic methanogenesis and the Wood–Liungdahl pathways are the two possible operational paths that can take place in an ideal situation [28]. In accordance with Equation (12), the direct conversion of CO_2_ to CH_4_ is carried out by hydrogenotrophic methanogenesis, which also involves the contribution of H_2_ as a source of electrons, whereas the Wood–Ljungdahl pathway includes two reactions in Equations (13) and (14) that indirectly convert CO_2_ to CH_4_ [28].

### 3.3. The BP System Bioenergy Estimation

The BP system was explored to recover bioenergy from its anaerobically digested wastewater as a beneficial option to offset the energy required by the UV-light. Generally, biogas constitute about 60–65% methane and 35–40% carbon dioxide [27], hence estimating 60% of 1 m^3^ biogas produced as energy is equivalent to 6 kWh. Herein, biogas as a renewable and sustainable energy source can be used to produce heat or electricity in place of fossil fuel [26,36,37]. In this study, the energy estimation is carried out to evaluate the amount of electricity that can be produced from the biogas generated and how much of it can be used to offset the UV-light power required. At 6 g Fe-TiO_2_ charged (Figure 3), a cumulative biogas of 0.00515 m^3^ yielded 90% methane (Figure 4) with energy content of 0.0464 kWh. From Table 3, assuming 75% of this energy can be converted into electricity resulted in 0.0348 kW/h. Subsequently, the total energy consumption for the UV-light to degrade the unit mass of pollutant above 80% (Figure 2) was estimated as 0.0180 kWh with net-energy of 0.0168 kWh. Also, the projected cost was estimated by adopting the basis and energy reports on biogas production from wastewater settings [26]. Herein, the energy cost per hour was found to be ZAR 0.054. From these estimations, it is determined that the BP system is energy-saving and cost-effective with a profit margin of $2.78 (ZAR 38.86) for 30 days.

### 3.4. Future Prospect of the BP System

Rapidly increasing water pollution due to the overwhelming discharge of an ever-increasing amount of different recalcitrant pollutants, combined with stricter environmental regulations, has resulted in a pressing need for the development of more efficient, commercially sustainable, and environmentally friendly water treatment technologies [2,3]. Thus, AD has been acknowledged as a successful process for converting vast amounts of organic waste from wastewater into biomethane [4,16]. However, AD produces large volumes of complex sludge, and the discharge of this sludge into the environment is a major source of environmental concern. Even though photocatalysis has the advantage of mineralizing resistant contaminants, it is also energy-intensive, making it a less desirable option. Development of the biophotocatalysis (BP) system, which involves the use of a biocatalyst (such as enzymes or microorganisms) in conjunction with photocatalysis, is a promising bioenergy technology that is currently in the early phases of exploration. Table 4 shows exemplary studies on biophotocatalysis for the removal of recalcitrant compounds. Notwithstanding, the use of the BP system demonstrated great potential to increase degradation efficacy of the wastewater above 80% COD removal. This resulted in an increase in the biogas yield and CH_4_ content. Because of this, the findings from this study will aid in compensating for the energy required when deploying the BP system in a real-world wastewater treatment setting. This will therefore assist in the paradigm shift of wastewater treatment towards water-energy benefits.

## 4. Materials and Methods

### 4.1. Wastewater Sample

**Figure 6:**
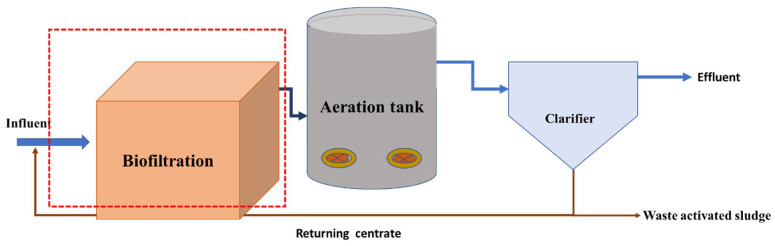
The block diagram of a local South Africa wastewater treatment plant with effluent sampled from the downstream of a biofiltration system.

The activated sludge and effluent sample were collected from a local South Africa municipality wastewater treatment plant based in the eThekwini municipality of the KwaZulu-Natal province. As shown in Figure 6, the effluent was collected at the downstream of the biofiltration system and was characterized by observing all standard procedures [43]. Table 5 shows the effluent characterized water parameters with their respective analytical instruments.

### 4.2. Magnetised Photocatalyst (Fe-TiO_2_)

The magnetized photocatalysts (Fe-TiO_2_) with a surface area of 62.73 m^2^/g used was inhouse engineered and characterized at the DUT, Chemical Engineering Research Lab, Steve Biko campus, S3L3, Durban, South Africa. The protocols used were in accordance with our previous work [26] and other studies [41,42]. All chemicals used, unless modified, were of analytical grade and obtained from Sigma Aldrich, South Africa. These included sodium hydroxide pellets (NaOH), ferrous sulphate heptahydrate (FeS0_4_·7H_2_O), ferrous chloride hexahydrates (FeCl_3_·6H_2_O) and titanium dioxide Degussa P-25 (anatase 70% and surface area of 25.7 m^2^/g).

**Figure 7:**
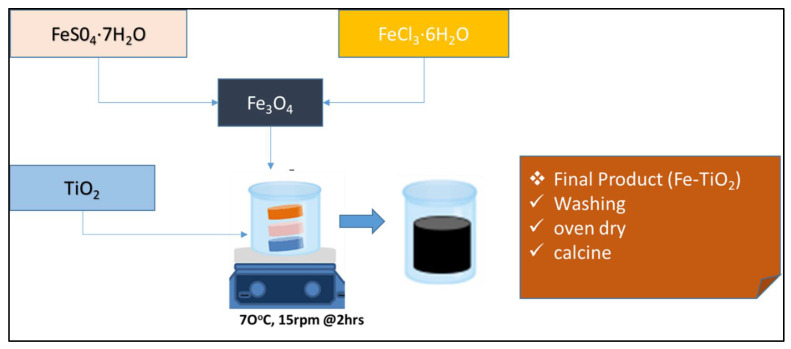
A schematic diagram of co-precipitation of magnetized photocatalyst (Fe-TiO_2_).

Figure 7 illustrates that the iron oxide nanoparticles (${\mathrm{Fe}}_{3}O_{4}$) were firstly formed from the aqueous Fe^2+^ and Fe^3+^ salt solutions by the addition of the NaOH (aid balance the pH) with the reaction expressed in Equation (15). Herein, the Degussa P25 TiO_2_ was then introduced into the solution and allowed for continuous stirring for 2 h until black precipitate was formed. The inclusion of Fe in the Fe-TiO_2_ expedited their paramagnetic phase transformation and charge reaction (16).

(15) $${\mathrm{FeSO}}_{4}\cdot 7H_{2}O+2{\mathrm{FeCl}}_{3}\cdot 6H_{2}O+8\mathrm{NaOH}\to {\mathrm{Fe}}_{3}O_{4}+6\mathrm{NaCl}+23H_{2}O+{\mathrm{Na}}_{2}{\mathrm{SO}}_{4}\;$$

(16) $${\mathrm{Fe}}^{3+}+{\mathrm{Ti}}^{4+}+2O^{2-}\to \left({\mathrm{Fe}}^{2+}+O^{2-}+\Delta_{a}\right)+{\mathrm{Ti}}^{3+}+\frac{1}{2}{\;O}_{2}$$

### 4.3. Biophotocatalytic (BP) System Description

**Figure 8:**
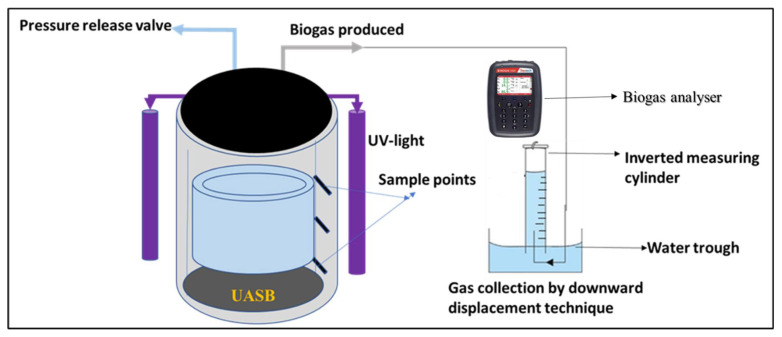
A schematic representation of the biophotocatalytic (BP) system consisting of the upflow anaerobic sludge blanket (UASB) reactor coupled with UV-light.

An upflow anaerobic sludge blanket (UASB) reactor constructed with Plexiglass coupled with UV-light bulbs (T8 blacklight-blue tube, 365 nm, 18 W, Philips, Amsterdam, Netherlands) was used as the BP system (Figure 8). The BP system was 60 cm in height and 20.7 cm in diameter with a total volume of 10 L and a working volume of 8 L with a headspace of 2 L. To reduce variations in temperature, continuous recycling of cooling water through the water jacket of the reactor was carried out using a water bath at constant temperature of 37.5 °C. Aside from the gas collection and purging port, there are sampling points which were used for the monitoring of the reactor performance with regards to effluent quality and sludge production, as well as desludging of the reactor. The BP system was run for four (4) weeks, and a weekly analysis of the water quality and biogas composition was done. The first week had no Fe-TiO_2_ addition, followed by the system being dosed with 2 g Fe-TiO_2_ every week until the fifth week when the system was shut down. However, a daily monitoring of the biogas produced was done via the downward displacement technique and the biogas composition was characterized with a Geotech Biogas 5000 Portable Biogas Analyzer (ISO17025). The contaminant removal percentage (%R) was evaluated using Equation (17).

(17) $$Reactor\;efficiency\;\left(\%R\right)=\left(\frac{C_{i}-C_{f}}{C_{i}}\right)\times 100$$

where, $C_{i}$ = Initial sample concentration and $C_{f}=$ final sample concentration.

To establish the rate of degradation, the cumulative biogas obtained was modelled and compared with the pseudo first order (18) and modified Gompertz (19) kinetic models [44,45].

(18) Y(t)=Ym [1−exp(−kt)]

(19) $$Y\left(t\right)=\mathrm{Ym}.\exp\left(-\exp\left[\frac{2.7183R_{\max.}}{\mathrm{Ym}}\left[\lambda -t\right]\right]+1\right)$$

where, Y(t) is cumulative of specific biogas yield (mL/g COD), Ym is maximum biogas production (mL/g COD), λ is lag phase period or minimum time to produce biogas (days), t is cumulative time for biogas production (days), R_max_ is the maximum specific substrate uptake rate (mL/g COD.day), and k is a first-order rate constant (1/d).

## 5. Conclusions

The rise of water pollution and stringent regulations necessitate cost-effective and environmentally friendly solutions. As a result, the advancing of biological technologies is to make full usage of biogas produced and its by-products, as well as mitigating any disposal to the landfills in the water sector. Herein, the cost of implementing a standalone system to improve water and biogas quality has been a major barrier for its industrial application. Therefore, this study explored the biophotocatalysis (BP) system involving biocatalytic and photocatalytic mechanisms for degradation of a local South Africa wastewater into biogas. The resulting experimental data showed incorporating magnetized photocatalysts (Fe-TiO_2_) with a surface area of 62.73 m^2^/g improved the water quality (>80% COD, color, and turbidity removal), biogas production (5150 mL) and CO_2_ sequestration into methane (90% CH_4_). The cost-energy benefits were estimated based on the amount of electricity produced by the BP system from the biogas to subsidize the UV-light power required. At 6 g Fe-TiO_2_ charged, cumulative biogas of 0.00515 m^3^ yielded 90% CH_4_ with an energy content of 0.0464 kWh. By assuming 75% of this energy, electricity of 0.0348 kW/h was estimated. The BP system was found to be energy saving with a subsidized energy cost-profit margin of $2.78 (ZAR 38.86) for 30 days. The BP system then proved to be a cost-effective technology, as the bioenergy produced was able to compensate for the energy required by the UV-light. This shows that the development of BP systems, which is a combination of a photocatalyst and a biocatalyst, can overcome the constraints associated with only using a photocatalytic or biocatalytic system. Therefore, prospects of the BP system applicability and viability in a large-scale wastewater treatment setting should be given attention with regard to wastewater treatment and its bioeconomy.

## Figures and Tables

**Table 1:** A summary of results obtained for the weekly catalyst load of Fe-TiO_2_ on organic contaminants by using the biophotocatalytic system for four weeks.

Parameters	Week 1	Week 2	Week 3	Week 4
Catalytic loading (g)	0	2	4	6
pH	5.59 ± 1.2	7.13 ± 1.4	7.13 ± 1.3	7.69 ± 1.2
COD (mg COD/L)	115 ± 3.4	87 ± 2.3	30 ± 2.1	12 ± 1.3
Total N (mg/L)	24.61 ± 1.4	7.26 ± 2.2	0.98 ± 0.15	2.65 ± 0.32
TKN (mg/L)	24.3 ± 1.3	6.01 ± 1.2	0.80 ± 0.1	2.53 ± 0.3
NO_3_-N (mg/L)	0.31 ± 0.1	1.25 ± 0.7	0.18 ± 0.05	0.12 ± 0.02
NH_4_^+^ (mg/L)	0.89 ± 0.13	0.78 ± 0.2	0.76 ± 0.12	0.74 ± 0.14
VS/TS	0.35	0.19	0.32	0.48

Chemical oxygen demand (COD), Total Nitrate, Total Kjeldahl nitrogen (TKN), Nitrate-Nitrogen (NO_3_-N), Ammonia (NH_4_^+^), Total solids (TS) and Volatile solids (VS).

**Table 2:** A summary of the modified Gompertz and first order kinetic models for the BP system.

Terms	Modified Gompertz Model	First Order Model
Ct (mL/g COD)	4780	4780
Cm (mL/g COD)	4830	3,141,923
k (1/day)	0.20623	0.00006
ʎ (day)	10.7	N/A
Sum of square errors (SSR)	1,152,385	7,258,214
Correlation Coefficient (R^2^)	0.9917	0.9351
Predicted yield (mL/g COD)	4741	5659

**Table 3:** The estimated energy cost of the BP system biogas produced from wastewater.

Item No	Item	Values
	*Biogas produced (m^3^)*	0.00515
1	Energy content of Methane (m^3^/h)	0.0464
2	Methane for electricity (kW/h)	0.0348
3	Energy applied (UV) (kW/h)	0.0180
4	Net energy (2–3) (kW/h)	0.0168
	*Cost estimation*	
5	Energy cost (3.22 ZAR/kWh)	0.054
	Energy cost (0.23 USD/kWh)	0.0039
6	*Net energy cost for 30 days*	
	Energy cost (3.22 ZAR/kWh)	38.86
	Energy cost (0.23 USD/kWh)	2.78

**Table 4:** Studies on recalcitrant compound degradation by the BP system.

Biocatalyst	Photocatalyst	Degradation Efficiency	Reference
Biofilm	SiO_2_-TiO_2_	100% phenol	[38]
Glucose oxidase	TiO_2_	>99% acid orange 7	[39]
Biofilm	Ag/TiO_2_	94% Tetracycline	[40]
Microcystis aerugionsa	Ag/TiO_2_	96% Tetracycline 75% Cr (VI)	[41]
GOx	NiFe_2_O_4_	98.6% Indigo carmine	[42]
Activated sludge	Fe-TiO_2_	>80% COD	This study

**Table 5:** The characterization of wastewater and activated sludge samples.

Water Quality	Value	Analytical Instrument
pH	7.4 ± 1.6	Hanna pH/EC/TDS Tester (H198130)
Temperature (°C)	26.4 ± 2.3	Hanna pH/EC/TDS Tester (H198130)
Colour (abs 465 nm, Pt.Co)	570 ± 7.6	HACH Spectrophotometer (DR3900)
Turbidity (NTU)	732 ± 12.5	Turbidity meter (HACH 2100N)
Chemical oxygen demand (mg COD/L)	2380 ± 32	HACH Spectrophotometer (DR3900)
Ammonia (mg NH_3_/L)	0.7 ± 0.2	HACH Spectrophotometer (DR3900)
Total Kjeldahl nitrogen (mg TKN/L)	30.5 ± 1.4	HACH Spectrophotometer (DR3900)
Nitrate (mg NO_3_/L)	0.6 ± 0.15	HACH Spectrophotometer (DR3900)
Total nitrogen (mg TN/L)	31.9 ± 1.8	HACH Spectrophotometer (DR3900)
Total suspended solids (mg TS/L)	304.5 ± 23.6	Analytical balance (HCB602H 22 ADAM)
Volatile solids (mg VS/L)	229.5 ± 2.65	Analytical balance (HCB602H 22 ADAM)
Ratio VS/TS	0.75	

## Data Availability

Not applicable.

## Sample Availability

Samples of the compounds are not available from the authors.
